# Zebrafish Models of Diamond-Blackfan Anemia: A Tool for Understanding the Disease Pathogenesis and Drug Discovery

**DOI:** 10.3390/ph12040151

**Published:** 2019-10-09

**Authors:** Tamayo Uechi, Naoya Kenmochi

**Affiliations:** Frontier Science Research Center, University of Miyazaki, 5200 Kihara, Kiyotake, Miyazaki 889-1692, Japan; t_uechi@med.miyazaki-u.ac.jp

**Keywords:** ribosome, Diamond-Blackfan anemia, zebrafish, disease model, drug candidate

## Abstract

Diamond-Blackfan anemia (DBA) is a rare bone marrow failure syndrome characterized by red blood cell aplasia. Currently, mutations in 19 ribosomal protein genes have been identified in patients. However, the pathogenic mechanism of DBA remains unknown. Recently, several DBA models were generated in zebrafish (*Danio rerio*) to elucidate the molecular pathogenesis of disease and to explore novel treatments. Zebrafish have strong advantages in drug discovery due to their rapid development and transparency during embryogenesis and their applicability to chemical screens. Together with mice, zebrafish have now become a powerful tool for studying disease mechanisms and drug discovery. In this review, we introduce recent advances in DBA drug development and discuss the usefulness of zebrafish as a disease model.

## 1. Introduction

Mutations in the genes encoding ribosome components and ribosome biogenesis factors lead to disease conditions called ‘ribosomopathies’ [1,2]. The patients commonly display hematopoietic failures, skin and skeletal abnormalities, and cancer predisposition. Currently, various ribosomal protein (RP) genes and ribosome biogenesis or assembly factor genes have been identified as the disease-causing genes (Table 1). Diamond-Blackfan anemia (DBA; MIM# 105650) is the first discovered and most widely studied ribosomopathy, in which mutations in as many as 19 RP genes have been identified [2,3,4]. Although extensive efforts have been made during the last two decades, the pathogenic mechanism of DBA remains poorly understood, and there are no clinically effective treatments available. This is because of the limited availability of patient samples and the lack of appropriate animal models, which is a common issue for ribosomopathies.

To overcome these problems, attempts have been made to develop various disease models that exhibit DBA phenotypes, including patient-derived cultured cells or iPS cells, knockdown or knockout mouse models of causative genes, and others [5,6,7,8]. However, patient cells are scarce and difficult to obtain. Additionally, it is rather difficult to recapitulate the complex patient phenotypes with cultured cells. Knockout mice harboring a homozygous mutation of the causative RP genes died in a very early stage of development, and the heterozygous mutants displayed no abnormal symptoms [7].

The zebrafish (*Danio rerio*) is a small freshwater fish originating from India and is a useful model animal for studying human diseases [9,10,11]. There are many attractive advantages of using zebrafish as a DBA model, including not only the fast development and transparency of embryos but also the conserved hematopoietic regulation in humans. In addition, various technologies are available to create disease models, such as the knockdown of a target gene using morpholino antisense oligonucleotides (MOs) or the knockout using a CRISPR/Cas9 system [12,13]. Valuable resources are also available. There is a large resource center at the University of Oregon called ZIRC (Zebrafish International Resource Center), where thousands of mutant lines are maintained and detailed information about phenotypes and relevant literature is provided.

For these reasons, zebrafish have been recognized as a promising animal model of DBA, and several groups, including us, successfully developed valuable zebrafish models of this disease [10,14]. These models enabled us to gain an in-depth understanding of the disease mechanism and to provide platforms for empowering future studies. In this review, we introduce recent progress in developing zebrafish DBA models and exploring drug candidates for DBA.

## 2. Clinical Features and Associated Genes

DBA is an inherited pure red cell aplasia and displays hypoplastic anemia with reduced erythroid progenitors in the bone marrow [15]. Approximately half of the patients also display congenital anomalies, including thumb deformity, webbed neck, cleft palate, and cardiac malformation. In addition, these patients have an increased risk of cancer development, such as acute myeloid leukemia, myelodysplastic syndrome, colon cancer, and osteogenic sarcoma [2]. Patients are generally diagnosed during infancy or early childhood with an incidence rate of approximately 7 per million live births.

Treatment with corticosteroids, such as dexamethasone, can improve the anemia phenotype in 80% of cases, which is the first-line treatment for DBA [15]. However, patients often lose their response over long-term corticosteroid therapy and become dependent on regular red blood cell transfusions, resulting in adverse iron overload that has to be managed through the life [16]. Hematopoietic stem cell transplantation, the only curative treatment of DBA, is also available, but this procedure is restricted to those with a matched related donor and still exhibits a considerable risk of mortality associated with the treatment. Thus, there is a strong demand for new therapeutics for DBA.

To date, heterozygous mutations in 19 RP genes (*RPS7*, *RPS10*, *RPS15A*, *RPS17*, *RPS19*, *RPS24*, *RPS26*, *RPS27*, *RPS28*, *RPS29*, *RPL5*, *RPL11*, *RPL15*, *RPL18*, *RPL26*, *RPL27*, *RPL31*, *RPL35*, *RPL35A*) have been identified in DBA patients [17,18]. These mutations account for ~70% of the mutations found in patients. In addition, mutations in *GATA1*, a hematopoietic master transcription factor gene necessary for proper erythroid production and mutations in *TSR2*, an RPS26 chaperone protein gene, were also identified in a small number of patients [19,20].

Various studies have shown that defects in ribosome biogenesis causes nucleolar stress that triggers a p53 signaling pathway, which highlights a possible role of p53 in DBA pathogenesis [21,22]. Several ribosomal proteins, including RPL5 and RPL11, have been identified as key players that mediate p53 responses through interactions with murine double minute 2 (MDM2), the negative regulator of p53. Analyses of mouse and zebrafish DBA models also implicated p53 as the major contributor to the pathophysiology of DBA (see later sections) [23,24]. However, it remains controversial whether the p53-dependent pathway directly links to an anemia phenotype in DBA [25,26]. In addition, the observation that *RPL5* and *RPL11* are frequently mutated in the patients challenges the role of p53 in DBA pathophysiology. Recently, it was proposed that ribosome defects caused by RP haploinsufficiency or its faulty assembly resulted in reduced translation of a subset of transcripts such as *GATA1* mRNA, thereby impeding erythroid lineage commitment in DBA [27,28]. Although the mechanism underlying such selective alterations of mRNA translation is not fully understood, it may explain the pathogenic mechanisms of ribosomopathies including DBA.

## 3. Mouse and Zebrafish DBA Models

### 3.1. Mouse Models

To understand the disease mechanisms and to evaluate novel therapies, the generation of animal models is crucial. The first attempt to create a mouse model for DBA was carried out by Matsson et al. in 2004 [7], where they disrupted the mouse ortholog of human *RPS19*, which is the most frequently mutated gene in DBA patients. However, this trial ended up with embryonic lethality for mice harboring homozygous loss of *Rps19* and a normal hematopoietic phenotype for mice harboring heterozygous loss of *Rps19*. Four years later, another mouse model of DBA was developed that carries a heterozygous missense mutation in *Rps19* (*Dsk3* mutant). However, unlike DBA patients, the *Dsk3* mutants showed only mild anemia and a more prominent pigmented phenotype without any physical deformities [24]. Furthermore, a transgenic mouse model expressing an *RPS19* missense mutation at codon 62 (RPS19R62W) demonstrated growth retardation and a mild anemia with a reduced number of erythroid progenitors [29]. However, this mutation was thought to act by a dominant negative mechanism, which is different from the generally believed haploinsufficiency model for DBA.

In 2011, Jaako et al. finally generated a mouse model of DBA with decreased erythrocyte production using transgenic RNA interference, where the synthesis of Rps19 was downregulated by a doxycycline-regulated *Rps19*-targeting shRNA [8]. The Rps19-deficient mice displayed macrocytic anemia, leukocytopenia, and variable platelet counts. Subsequently, Morgado-Palacin et al. generated mice with an inducible *Rpl11*-null allele and demonstrated that heterozygous loss of *Rpl11* in adult mice impaired erythrocyte maturation [30]. Interestingly, these mice also showed reduced p53 responses and increased cMYC levels. Together, these defects resulted in anemia and cancer susceptibility, thereby recapitulating DBA. *Rps19* knockdown and *Rpl11* haploinsufficient mice proved to be useful, but they have not become widely used due to their limited availability. Moreover, attempting high-throughput exploration of drug candidates using such mouse models is generally difficult.

### 3.2. Zebrafish Models

**Figure 1:**
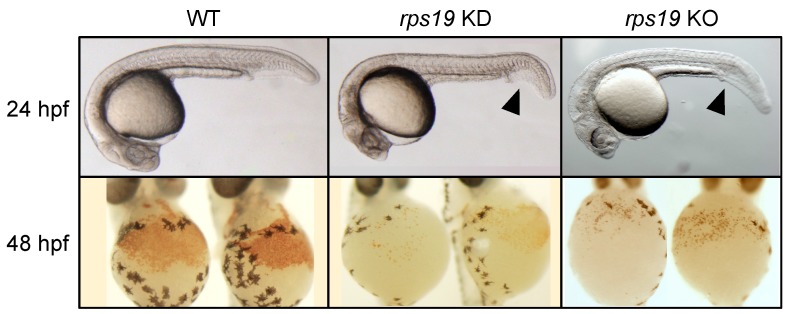
Zebrafish DBA models. Wild-type embryos show a high density of red blood cells (WT), whereas the blood production is drastically reduced in the *rps19* knockdown (KD) and knockout (KO) embryos, as indicated by the absence of hemoglobin stained cells (lower panels). These embryos also display developmental deformities in head and tail regions (arrowheads). The abnormal phenotypes were rescued by injection of *rps19* mRNA but not rescued by mRNA with patient type mutations in the knockdown embryos [14].

To compensate for the difficulties and problems encountered by the mouse models, we and other groups focused on zebrafish for more convenient and high-throughput analyses. Knockdown of zebrafish *rps19* nicely recapitulated DBA phenotypes [14,23,25], which displayed a drastic reduction in red blood cells (Figure 1). Interestingly, as observed in DBA patients, only erythroid cell differentiation was affected, but other myeloid and endothelial cells were unaffected. The knockdown embryos also showed deformities in the head and tail regions, which may be relevant to the skeletal abnormalities observed in the patients. These phenotypes were rescued by injection of wild-type *rps19* mRNAs but not by mRNAs with patient-type mutations [14]. Subsequently, other RP gene knockdown models were also generated, and together with the rps19-knockdown embryos, these models have been used for exploring DBA pathogenesis [10].

As described above, a role of p53 signaling pathway is controversial. Danilova et al. showed that both developmental abnormalities and erythroid reduction were caused by activation of p53 [23], whereas Torihara et al. demonstrated that only developmental abnormalities are dependent on the p53 pathway but erythroid defect was independent, which was shown by a simultaneous knockdown of *rps19* and *tp53* in zebrafish [25]. Similarly, p53-inependent erythropoiesis failure was demonstrated in *rpl35a* and *rps24* knockdown embryos using *tp53* mutant zebrafish [31]. In contrast, a knockdown of *rps7* was somewhat complicated, which exhibited both p53 dependent and independent features [32].

Valuable zebrafish mutant lines have also been developed. Amsterdam et al. generated several hundred lines of zebrafish, each heterozygous for a recessive embryonic lethal mutation, by insertional mutagenesis. These include RP gene mutants and surprisingly many of them developed malignant peripheral nerve sheath tumors, suggesting that many RP genes are cancer genes in zebrafish [11]. Subsequently, Taylor et al. found that one of these mutants, *rps29*^-/-^, displayed decreased red blood cell production and increased apoptosis in the head region during early development [33]. Both the hematopoietic and apoptotic phenotypes were rescued by p53 inactivation, which is in good agreement with a speculated role of p53 in the pathogenic mechanism of DBA. Similarly, *rpl11* mutant identified from the same mutagenesis screen showed anemia, decreased hematopoietic stem cells, and activation of the p53 pathway with altered expression in genes involved in cell cycle arrest and apoptosis [34].

Recently, the progress of genome editing technologies, such as the TALEN and CRISPR/Cas9 systems, enabled us to develop zebrafish mutants more rapidly and precisely. Zhang et al. successfully generated an *rps19* null mutant through TALEN-mediated gene targeting, which reproduced the erythroid defects of DBA, including a lack of mature red blood cells, decreased globin synthesis, and p53 activation [35]. Our group also generated several *rps19* mutants that display decreased erythroid production by using the CRISPR/Cas9 system (Figure 1, unpublished data). All these mutants are useful resources for DBA research, especially for in vivo drug screening and evaluations of the efficacy.

## 4. Drug Development

**Figure 2:**
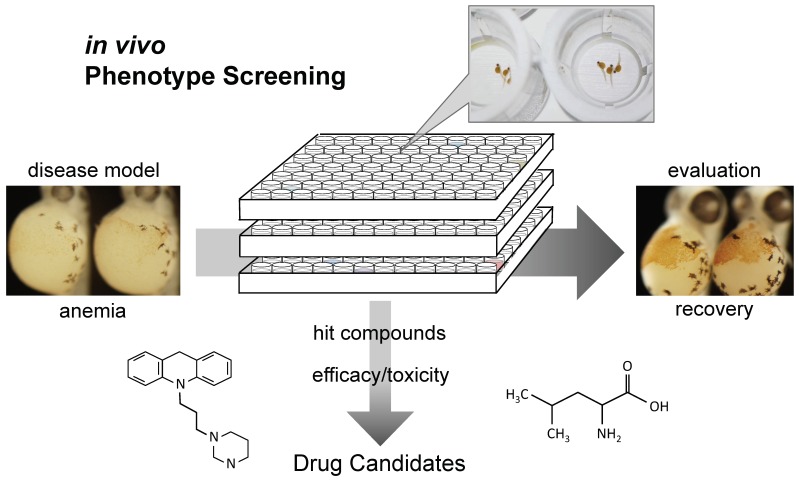
Zebrafish in vivo phenotype screening. Zebrafish provides a novel platform for screening compound libraries and evaluating drug efficacy in vivo, which will lead to find new therapeutics for rare diseases including DBA.

Corticosteroid treatments and chronic transfusion are the main therapeutic regimens in DBA. Although these therapies can alleviate anemia, both cause severe long-term toxicities. To develop new therapeutics for DBA, zebrafish provide a powerful tool for evaluating drug efficacy and screening compound libraries (Figure 2, Table 2).

### 4.1. l-leucine

The amino acid l-leucine was the first molecule found to improve anemia in a zebrafish model of DBA. Payne et al. treated *rps19*-knockdown zebrafish with l-leucine and found that it improved the anemia and developmental defects associated with DBA [36]. These findings were confirmed in primary human CD34^+^ cells after shRNA knockdown of *RPS19*. l-Leucine was also effective in a mouse model of DBA. Administration of l-leucine significantly improved anemia in Rps19-deficient mice, showing increased erythrocyte numbers and hemoglobin concentration, and associating with decreased p53 activity [37]. Because erythroid progenitor cells have a very high rate of proliferation and require a high level of ribosome biogenesis, the erythroid lineage may be particularly sensitive to reduced expression levels of ribosomal proteins. On the other hand, l-leucine is thought to boost translation by upregulating ribosome biosynthesis through the mTOR pathway [38]. This may explain why l-leucine is effective in zebrafish and mouse models of DBA. A pilot phase I/II study of l-leucine in the treatment of patients with transfusion-dependent DBA is in progress under ClinicalTrials.gov with the number NCT01362595.

### 4.2. Sotatercept

Sotatercept (ACE-011) is an activin receptor type IIA ligand trap that inhibits transforming growth factor beta (TGF-β) superfamily members, including growth differentiation factor 11 (GDF-11) and activin B [39]. It was generated by recombinant fusion of the extracellular domain of the human activin receptor IIA and the human immunoglobulin G1 Fc domain. GDF-11 is overexpressed in immature erythroblasts in β-thalassemia, and an activin receptor IIA ligand trap was able to correct ineffective erythropoiesis in patients [40]. Ear et al. investigated the ability of the murine ortholog of sotatercept (RAP-011) to restore erythroid levels in zebrafish models of DBA, including *rps19* and *rpl11* knockdown embryos and to elucidate the role of TGF-signaling molecules during ribosome stress [41]. They demonstrated that RAP-011 was able to elevate erythroid counts under normal physiological conditions and during ribosome deficiency in zebrafish. In addition, RAP-011 was shown to block the function of lefty1 (Lft1), an activin/nodal signaling antagonist, and its overexpression delayed the maturation of erythroid cells. In this context, increased expression of Lft1 was observed in *rps19* and *rpl11*-mutant zebrafish. A safety and efficacy study of sotatercept in adults with transfusion-dependent DBA is in progress under ClinicalTrials.gov with the number NCT01464164. 

### 4.3. Trifluoperazine

A chemical screen using zebrafish *rps29*^-/-^ embryos identified several calmodulin (CaM) inhibitors that can rescue hemoglobin levels in mutant embryos [42]. Subsequently, the effects of the CaM inhibitor trifluoperazine (TFP) were examined in human and murine models. Treating *RPS19*-deficient cord blood-derived CD34+ cells with TFP relieved the erythroid differentiation block, and injection of TFP into an Rps19-deficient mouse model ameliorated erythroid production and hemoglobin level. In addition, TFP was suggested to inhibit p53 transcriptional activity through its c-terminal domain (CTD), thereby rescues the phenotypes caused by RP deficiency [43]. CaM inhibitors may be effective therapies for DBA patients. A phase I/II study to determine the safety of TFP in transfusion-dependent DBA has just started and can be found at ClinicalTrials.gov with the number NCT03966053.

### 4.4. SMER28

Screening of a chemical library using iPS cells from DBA patients identified SMER28, an inducer of autophagy, as a hit compound that enhances erythroid differentiation of iPS cells [44]. These cells were established from the fibroblasts of patients with *RPS19* and *RPL5* inactivating mutations. To assess the therapeutic potential of SMER28 in vivo, a zebrafish model of DBA was tested. Treatment with SMER28 improved erythroid production in *rps29*^-/-^ zebrafish in a dose-dependent manner. More than 50% of embryos treated with 1 μM SMER28 showed high expression of hemoglobin compared to 20% for the controls. SMER28 was also examined its effects on mice with irradiation-induced anemia, which resulted in elevated RBC counts and hematocrit. Furthermore, the effects of SMER28 were tested in *Rpl11*^+/fl^/Mx1-Cre mice that displayed decreased erythropoiesis, in which deletion of *Rpl11* was under the control of poly(I:C)-inducible Cre. The poly(I:C)-induced anemic mice receiving SMER28 (20 mg/kg) showed significant improvement of RBC counts [44]. Because the effects of SMER28 on human cells have been confirmed as well, SMER28 or its derivatives may be viable alternatives to current treatments for DBA. Clinical studies on the safety and efficacy of SMER28 are needed. 

## 5. Conclusions

Zebrafish DBA models were generated and have now become powerful tools for studying DBA pathogenesis. More importantly, zebrafish have made in vivo chemical screening possible, which will lead to the identification of compounds that are effective for DBA therapies (Figure 2). L-Leucine, sotatercept, and trifluoperazine, identified using zebrafish DBA models, are now under evaluation in clinical trials. The divergent pathways illuminated by these chemicals also shed light on the pathophysiology of DBA. Zebrafish in vivo phenotype screening followed by validation of efficacy in mouse models will be a promising strategy to develop new therapeutics for rare diseases such as DBA.

## Figures and Tables

**Table 1:** Ribosomopathies and causative genes [1,2].

Disease	Gene	Function	Abnormal Phenotypes			
			Blood	Tumor	Skin	Skeleton
Diamond-Blackfan anemia (DBA)	*RPS19 and other* *18 RP genes*	ribosomal protein	++	+		
X-linked dyskeratosis congenita (DC)	*DKC1*	rRNA modification enzyme	++	+	++	
Cartilage-hair hypoplasia (CHH)	*RMRP*	cleavage of 5.8S rRNA	++	+	++	++
Shwachman-Diamond syndrome	*SBDS*	promoting of 60S subunit maturation	++	+		++
T-cell acute lymphoblastic leukemia (T-ALL)	*RPL5, RPL10, RPL22*	ribosomal protein	++	++		++
5q- syndrome	*RPS14*	ribosomal protein	++	+		
Treacher-Collins syndrome	*TCOF1, POLR1D,* *POLR1C*	rDNA transcription				++
Isolated congenital asplenia	*RPSA*	ribosomal protein				

Note: Ribosomopathies are diseases caused by mutations in the genes encoding ribosomal proteins or RNAs or other ribosome biogenesis factors. They are characterized by hematologic and developmental disorders and often comprise bone marrow failure, skeletal and other developmental abnormalities and cancer predisposition. The mechanism by which a mutation in any ribosomal component results in hematologic disorder is still unclear, although the ribosome widely exists throughout the body.

**Table 2:** Drug candidates for DBA.

Compound	Function	Disease Model	Clinical Trial, Patent
L-Leucine [36,37,38]	activator of protein synthesis	hCD34+, zebrafish, mouse	NCT01362595
Sotatercept [39,40,41]	activin receptor type II ligand trap	zebrafish	NCT01464164
Trifluoperazine [42,43]	calmodulin inhibitor	hCD34+, zebrafish, mouse	NCT03966053
SMER28 [44]	inducer of autophagy	iPSC, zebrafish, mouse	N/A
CDK8 inhibitor [45]	inhibitor of cyclin-dependent kinase 8	mouse fetal liver cell	WO2017076968Al
Dimethyloxalylglycine * [46]	prolyl hydroxylase inhibitor	mBFU-E	N/A
GW7647 *, fenofibrate * [47]	PPAR-a agonist	mBFU-E, hCD34+, mouse	N/A

Note: * combination with glucocorticoid. PPAR-a, peroxisome proliferator-activated receptor a; hCD34+, human CD34+ cells; mBFU-E, mouse burst-forming unit erythroid.
